# Rho/SRF Inhibitor Modulates Mitochondrial Functions

**DOI:** 10.3390/ijms231911536

**Published:** 2022-09-29

**Authors:** Pankaj Patyal, Bachkhoa Nguyen, Xiaomin Zhang, Gohar Azhar, Fathima S. Ameer, Ambika Verma, Jasmine Crane, Grishma KC, Yingni Che, Jeanne Y. Wei

**Affiliations:** Donald W. Reynolds Department of Geriatrics and Institute on Aging, University of Arkansas for Medical Sciences, Little Rock, AR 72205, USA

**Keywords:** CCG-1423, serum response factor, mitochondrial function, acetylation

## Abstract

CCG-1423 is a Rho A pathway inhibitor that has been reported to inhibit Rho/SRF-mediated transcriptional regulation. Serum response factor and its cofactors, which include ternary complex factors and myocardin-related transcription factors, regulate various cellular functions. In this study, we observed that CCG-1423 modulates the mitochondrial functions. The effect of this small molecule drug was determined by measuring mitochondrial function using an XFe96 Analyzer and an Oxygraph 2k (O2k) high-resolution respirometer. CCG-1423 treatment significantly reduced oxidative phosphorylation in a dose-dependent manner. However, CCG-1423 increased the glycolytic rate. We also observed that histone 4 at lysine-16 underwent hyperacetylation with the treatment of this drug. Immunolabeling with F-actin and MitoTracker revealed the alteration in the actin cytoskeleton and mitochondria. Taken together, our findings highlight a critical role of CCG-1423 in inhibiting the transcription of SRF/p49 and PGC-1α, β, resulting in the downregulation of mitochondrial genes, leading to the repression of mitochondrial oxidative phosphorylation and overall ATP reduction. This study provides a better understanding of the effects of CCG-1423 on mitochondria, which may be useful for the assessment of the potential clinical application of CCG-1423 and its derivatives.

## 1. Introduction

Serum response factor (SRF) is an important transcription factor that plays a crucial role in multiple biological processes in many cells, such as muscle cells (cardiac, skeletal, and smooth), endothelial cells, fibroblasts, hepatocytes, immune cells, and neurons [1,2,3]. SRF regulates differential gene expression through cofactor(s) recruitment, among which the most well-known are myocardin-related transcription factors and the members of the ternary complex factors. Myocardin family transcriptional co-activators include myocardin, also known as MKL (megakaryoblastic leukemia), which plays an important role in stimulating transcriptional activity of SRF. In the nucleus, different cofactors associate with SRF, and this association drives gene expression via the CC(A/T-rich)_6_GG cis-element, also called the CArG box [4]. This complex regulates actin cytoskeleton and motility [5,6]. SRF is also regulated by Rho GTPases and the Rho/SRF signaling has also been studied in terms of mechanisms including melanoma metastasis and fibrotic pathological pathways [7,8,9].

A small molecule screen of inhibitors of Rho-induced SRF-mediated transcription initially discovered CCG-1423 as an inhibitor of MKL/SRF signaling. CCG-1423 is formally named as N-[2-[4(4-chlorophenyl) amino]-1-methyl-2-oxoethoxy]-3, 5-bis (trifluoromethyl)-benzamide [10]. It has been shown that CCG-1423 binds to the nuclear localization signal region of G-actin-binding sites of the MKL family of proteins [11]. CCG-1423 prevents MKL’s association with importin-α/β, causing inhibition of nuclear import of MKL and MKL/SRF-mediated gene transcription. CCG-1423 can also indirectly reduce the nuclear accumulation of MKL and repress SRF activation. Others have shown that the inhibitory effects of CCG-1423 on cancer cell invasions [10] tend to improve other pathological changes including insulin resistance [12], neointima formation in vascular diseases [13], and fibrogenesis [14].

It is evident that dysfunction in mitochondrial metabolism is the cause of several inherited and acquired diseases. Mitochondria are essential organelles that have several critical functions in the cell, including metabolism and signaling [15]. SRF regulates cell metabolism by maintaining mitochondrial dynamics, fatty acid translocation, and determining the fate of electron transport chain (ETC) complex proteins [16]. Epigenetic modifications are major components affecting the activation of genes downstream of SRF [17]. Chromatin structure and function are greatly influenced by histone protein post-translational modifications, and they usually regulate the DNA transcription factor binding by altering either acetylation or methylation. SRF is involved in mechanisms controlling chromatin structure [18]. The reversible addition of an acetyl group to lysine residues and protein acetylation regulates the transcriptional activity [19,20]. Furthermore, many studies have demonstrated the crucial role of SRF in cellular migration and normal actin cytoskeleton and contractile biology [21,22]. Previously, our group had discovered p49/STRAP, a serum response factor binding protein [23]. The NADH dehydrogenase ubiquinone oxidoreductase subunit AB1 (NDUFAB1), a subunit of complex I, interacts with p49/STRAP [24]. Moreover, p49/STRAP over-expression promoted histone H4 deacetylation, which was accompanied by downregulation of peroxisome proliferator-activated receptor gamma coactivator 1-alpha (PGC 1α) and mitofusin 1 and 2 expression [25]. Mitofusins, which are regulated by PGC-1α, are important mitochondrial fusion proteins [26]. Therefore, p49/STRAP has been demonstrated to have its role in mitochondrial function and dynamics.

We herein investigated the role CCG-1423 on SRF and its cofactor p49/STRAP and whether it repressed the mitochondrial function and biogenesis in mouse skeletal myoblast cells. Our data demonstrate that CCG-1423 deacetylated the SRF protein and caused histone 4 at lysine 16 (H4K16) hyperacetylation. It also changed the actin cytoskeleton conformation. In addition, it reduced the transcriptional activity of *PGC-1α*, *PGC-1β*, and affected the other mitochondrial genes involved in biogenesis and function. We have shown the effects of CCG-1423 on the functional aspects of mitochondrial function of oxidative phosphorylation (oxygen consumption rate, OCR), glycolysis (extracellular acidification rate, ECAR), and total ATP production. These findings help to elucidate the role of CCG-1423 in mitochondrial function and might suggest areas for future study in the preclinical evaluation of CCG-1423 as a potential novel therapeutic agent.

## 2. Results

### 2.1. CCG-1423 Downregulates SRF, p49/STRAP Expression and Does Not Affect the Cell Viability

**Figure 1:**
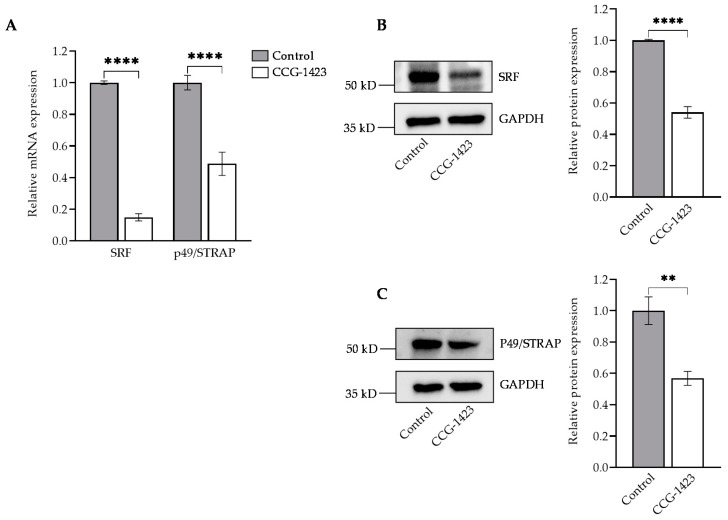
CCG-1423 represses SRF and p49/STRAP. (**A**–**C**) C2C12 cells were treated with a 10 µM dosage for 24 h. (**A**) The mRNA expression of SRF and p49/STRAP was evaluated in qPCR. DMSO was used as control. Immunoblotting was used to evaluate the protein levels of (**B**) SRF and (**C**) p49/STRAP. GAPDH was used as the total protein loading control and relative protein expression was quantified in (**B**,**C**). ** *p* < 0.01, **** *p* < 0.0001 (*n* = 3).

We first examined whether CCG-1423 affected C2C12 cell viability. The treatment of CCG-1423 at different concentrations for 24 h did not significantly affect the C2C12 cell viability. At higher concentrations, 1 μM and 10 μM of CCG-1423 did have a slight effect on the cell viability (Supplementary Figure S1). The inhibitory effects of CCG-1423 on SRF and p49/STRAP was tested at 10 μM dosage of CCG-1423 for 24 h, resulting in significant reduction of mRNA expression levels and protein levels of SRF and p49/STRAP (Figure 1A,B). Thus, CCG-1423 downregulates both SRF and p49/STRAP.

### 2.2. CCG-1423 Increases H4 Lysine-16 Acetylation but Decreases the Level of Acetylated-Lysine SRF

**Figure 2:**
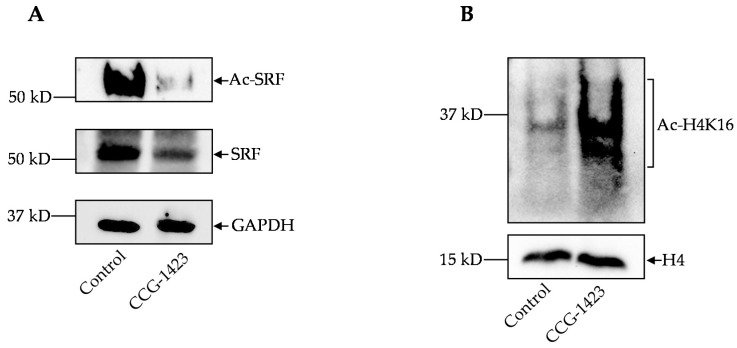
Immunoprecipitation of SRF acetylated lysine and H4K16 protein. (**A**) CCG-1423 represses the SRF acetylated-lysine (Ac-SRF), representative western blot of acetylated-lysine SRF and SRF in lysates from CCG-1423 treated and non-treated C2C12 cells. GAPDH was used as a loading control. (**B**) H4K16 was hyperacetylated on the CCG-1423 treatment, representative western blot image of H4K16 and total histone H4 protein was used as a loading control (*n* = 3).

We explored a possible regulation of SRF by CCG-1423 in immunoprecipitation experiments using an SRF antibody specifically pulled down SRF compared with vehicle control after 24 h, 10 μM dosage treatment. The strong signal of SRF pulldown confirmed the specificity of the SRF antibody (Figure 2A). The immunoprecipitation evidence for acetylated-lysine/SRF shows SRF is deacetylated on the treatment of CCG-1423 (Figure 2A). We then determined acetylation levels of H4K16 since it plays an important role in transcription and chromatin packaging [27]. We performed immunoprecipitation to specifically pull down the H4. Interestingly, our data showed that pre-treatment with CCG-1423 robustly increased H4K16 acetylation (Figure 2B).

### 2.3. CCG-1423 Represses the Genes Involved in Mitochondrial Biogenesis, Fusion, and Fission

**Figure 3:**
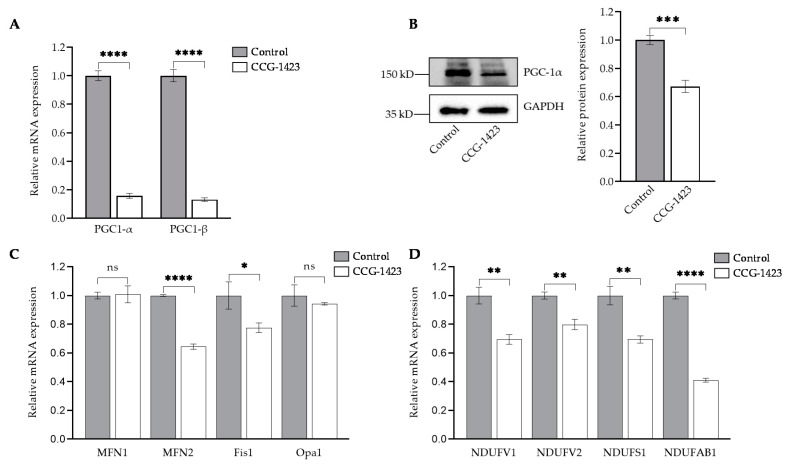
Repression of mitochondrial genes involved in biogenesis and function. (**A**) RT-qPCR analysis demonstrated the inhibition of *PGC-1α* and *-1β* gene expression following the treatment of CCG-1423. (**B**) Protein levels of PGC-1α were analyzed by Western blot and GAPDH was used as a loading control. Densitometric analysis shows inhibition of PGC-1α. (**C**) *MFN2* and *Fis1* gene expression levels reduced whereas *MFN1* and *Opa1* were unaffected. (**D**) Downregulation of mitochondrial genes involved in complex-I. * *p* < 0.05, ** *p* < 0.01, *** *p* < 0.001, **** *p* < 0.0001, ns: *p* > 0.05 (*n* = 3).

We next assessed the effect of CCG-1423 on the gene expression of *PGC-1α* and *PGC-1β*. The *PGC-1α* and *-1β* are the upstream regulators of mitochondrial genes [28,29]. Our data indicate that CCG-1423 reduced the *PGC-1α* and *PGC-1β* expression at mRNA levels (Figure 3A) as well as PGC-1α protein levels (Figure 3B). We also determined the effect of CCG-1423 on mitochondrial fusion and fission genes. CCG-1423 significantly reduced the expression level of mitofusin-2 (*MFN2*) and mitochondrial fission 1 (*Fis1*) whereas it did not change the mRNA expression level of mitofusin-1 (*MFN1*) and optic atrophy-1 (*Opa1*) (Figure 3C). CCG-1423 also altered the expression levels of mitochondrial complex I subunit genes *NDUFV1*, *NDUFV2*, *NDUFS1,* and *NDUFAB1* (Figure 3D).

### 2.4. Actin Assembly and Mitochondrial Morphology

**Figure 4:**
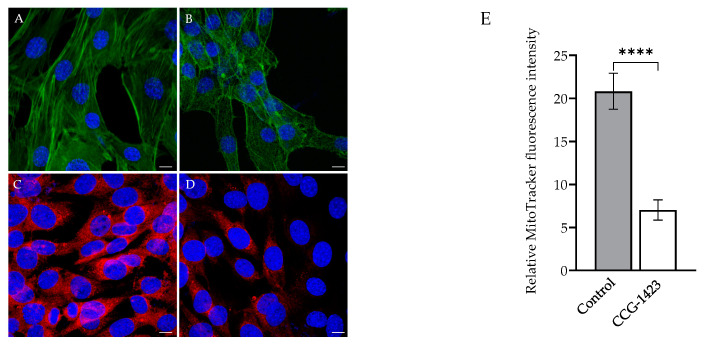
Immunolabeling of Actin and MitoTracker. C2C12 cells at 24 h post-treatment, cells incubated with phalloidin (green) and MitoTracker (red) conjugated dyes. DAPI (blue) was used for nuclear counterstaining. (**A**,**C**) Vehicle (DMSO) (**B**,**D**) CCG-1423 treated cells, at 10 μM dosage. 63× oil objective is used; scale bars indicate 10 μm. (**E**) MitoTracker stained cells are randomly selected microscopic fields in four individual repetitions where the fluorescence intensity was quantified using ImageJ. **** *p* < 0.0001 (*n* = 4).

SRF is a crucial transcription regulator of normal actin cytoskeleton and cellular migration [30]. The f-actin phalloidin staining for actin filaments traversing the entire length of the cells (in Figure 4A) were altered by CCG-1423 treatment (in Figure 4B). SRF inhibition decreased the f-actin formation and changed the cell structure. To further investigate the effect of CCG-1423 on mitochondrial morphology and membrane potential, the MitoTracker staining was performed. As shown in Figure 4C (vehicle control) and Figure 4D (treated), CCG-1423 reduced the mitochondrial size and lowered the mitochondrial membrane potential.

### 2.5. CCG-1423 Differentially Regulates Cellular Bioenergetics

**Figure 5:**
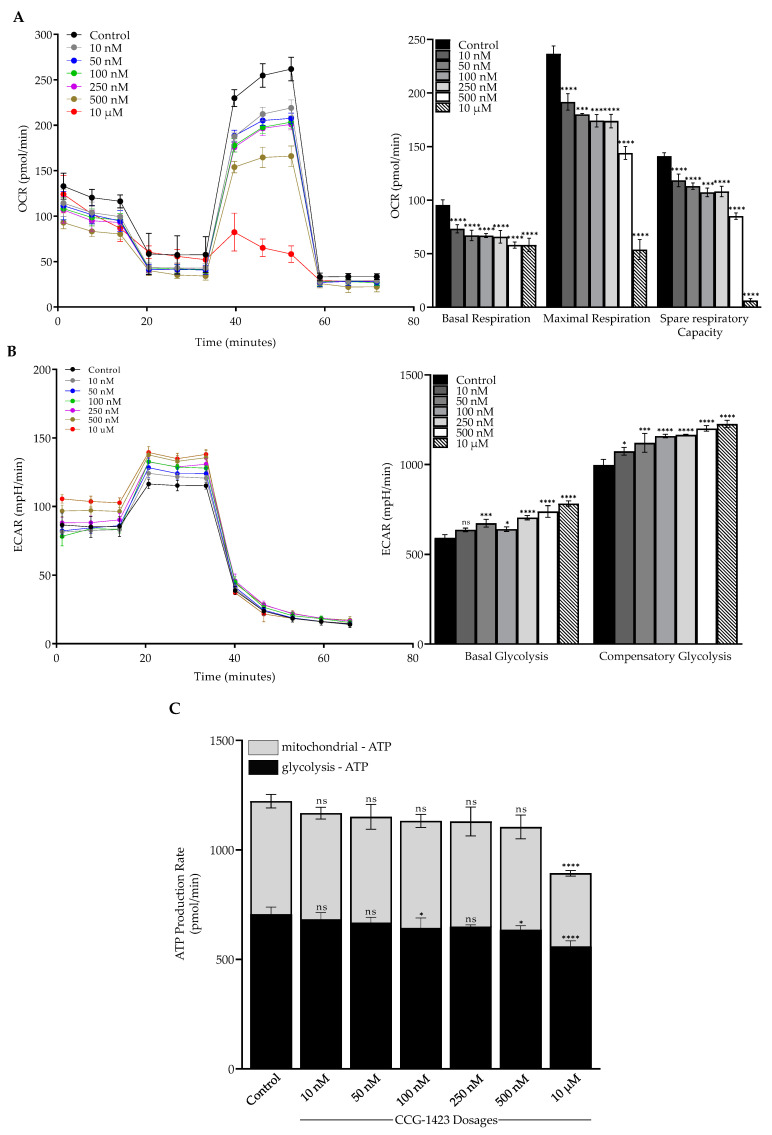
Mitochondria functional analysis of oxygen consumption rate, extracellular acidification rate, and total ATP production. (**A**) OCR and basal respiration, maximum respiration, and spare respiratory capacity in CCG-1423 treated cells with different dosages. OCR was repressed in a dose dependent manner upon the treatment of CCG-1423 in C2C12 cells. (**B**) CCG-1423 upregulated the ECAR in a dose dependent manner. (**C**) Total ATP production was decreased in both mechanisms of oxidative phosphorylation (mitochondrial-ATP) and glycolysis (glycolysis-ATP). * *p* < 0.05, *** *p* < 0.001, **** *p* < 0.0001, ns: *p* > 0.05 (*n* = 4).

To investigate the two major energy pathways, oxidative phosphorylation, and glycolysis, the Seahorse XFe96 Analyzer was utilized. CCG-1423 repressed the basal respiration paralleled by a significant reduction in the maximal respiratory capacity and spare respiratory capacity in a dose-dependent manner as compared to the vehicle control (Figure 5A). CCG-1423 increased the basal glycolysis and compensatory glycolysis in a dose-dependent trend (Figure 5B). Furthermore, we also determined the total ATP production from OCR and ECAR. As shown in Figure 5C, mitochondrial ATP was reduced with the treatment of different dosages of CCG-1423. The inhibitory effects on OCR reduced the mitochondrial ATP production, possibly forcing the cells to compensate by increasing glycolysis to meet energy demands.

**Figure 6:**
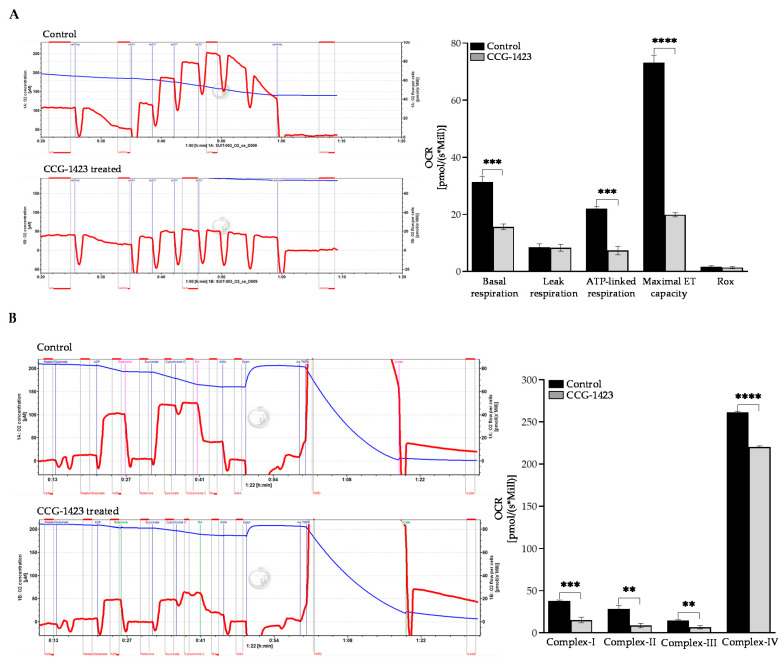
High-resolution respiratory analysis of oxidative phosphorylation. (**A**) OCR and basal respiration, leak respiration, ATP-linked respiration, Maximum ET capacity was analyzed using an Oroboros O2K instrument. Intact C2C12 cells were pre-treated with CCG-1423 with 10 µM for 24 h treatment. OCR and other functional aspects indicated above in the figure were repressed. (**B**) C2C12 cells were permeabilized and were used to determine OCR levels at complexes of ETC. OCR at complex I–IV of ETC were repressed on the treatment of CCG-1423. ** *p* < 0.01, *** *p* < 0.001, **** *p* < 0.0001 (*n* = 3).

Next, we tested the inhibitory effects of CCG-1423 on OCR with Oxygraph 2k high-resolution respirometer in the intact C2C12 cells. As shown in Figure 6A, the basal respiration, ATP-linked respiration, as well as the maximal capacity of the mitochondrial ETC was significantly repressed with the treatment of CCG-1423. With the substrate-inhibitor-titration, we also assessed the inhibitory effects on the Electron transport complexes I (NADH/ubiquinone oxidoreductase), II (succinate dehydrogenase), III (cytochrome c reductase), and IV (cytochrome c oxidase). Intriguingly, CCG-1423 represses the functional activity of all four complexes of ETC (Figure 6B).

## 3. Discussion

Recent advances in elucidating the gene transcriptional mechanisms downstream of Rho (i.e., MKL and SRF) have led to the discovery of CCG-1423, a first-generation inhibitor of Rho/MKL1/SRF-mediated gene transcription [10]. Since its initial discovery, a novel small-molecule inhibitor, CCG-1423, has demonstrated significant promise in various preclinical disease models, as an anti-fibrotic agent [14], a possible therapeutic agent in type II diabetes [12], an anti-angiogenic agent [31], and most importantly, in metastatic cancer [32,33,34,35,36]. 

CCG-1423 has been shown to inhibit the transcriptional signaling by RhoA GTPase family and inhibit proteins and genes, such as β-catenin, TAZ, and p-LATS1, which are involved in promoting proliferation of cancer cell lines. Interestingly, growing evidence suggests a major interest in pursuing the study of mitochondria biology in cancer and targeting this organelle therapeutically [37]. There are multiple ongoing clinical trials exploring the therapeutic effects of Oxidative Phosphorylation (OXPHOS) inhibitors in different tumor types [38]. Many preclinical studies have suggested anti-tumor activity of ETC CI inhibitors, for instance, metformin, the most common anti-diabetic drug [39,40,41]. Previously, our group has also demonstrated the role of metformin against low glucose-induced elevated oxygen consumption in a process that may involve reducing oxidative stress [42].

Although CCG-1423 is a pleiotropic drug, the effects of this small molecule inhibitor on mitochondria function are largely unknown. Our study is focused on exploring whether CCG-1423 could be utilized to target mitochondria metabolism. To address this gap in knowledge, we first defined the effect of CCG-1423 on the cell viability of C2C12 cells. As shown in Supplementary Figure S1, MTS assay demonstrated that incubation with different dosages of the drug molecule from 10 nm to 10 μM for 24 h did not affect the cell viability of C2C12. Our observation is consistent with no effect of the same dosage and for an extended period of 72h of CCG-1423 on cortical neurons [43]. Furthermore, other studies have also implicated the dose dependent inhibitory effects on SRF-mediated transcription by CCG-1423 and it has been shown that high doses of CCG-1423 (5 to 10 μM) are necessary for the reduction of smooth muscle α-actin expression at the protein levels [44]. The precise molecular mechanism of actions of CCG-1423 is not fully understood, but our findings indicate that CCG-1423 may disrupt Rho signaling through functional inhibition of SRF transcriptional activity (Figure 1A). Our data also suggest the inhibitory effects of CCG-1423 on the cofactor p49/STRAP, along with SRF (Figure 1A, B). The attenuation of MKL/SRF signaling is one of the main downstream effects of CCG-1423, but importantly our data suggest CCG-1423 has more than one cellular target. SRF binds to the serum response element in the promoter region of target genes and participates in cell cycle regulation, apoptosis, cell growth, and cell differentiation [45,46,47]. Our data indicates that CCG-1423 repressed the SRF acetylation at lysine (Figure 2). This was an expected outcome since many studies have shown the role of histone acetylation in SRF signaling [48]. Histone acetylation is a critical epigenetic modification that changes chromatin architecture and regulates gene expression by opening or closing the chromatin structure [49]. It has been studied that anisomycin and TNFα induce H4 hyperacetylation via the SAPK/JNK pathway [50]. CCG-1423 inhibition of SRF/p49 mediated transcription induces H4K16 hyperacetylation (Figure 2). Furthermore, histone hyperacetylation has been linked to inducing mitochondrial dysfunction [51,52].

*PGC-1α* and *β* are critical regulators of transcriptional control of mitochondrial biogenesis [25], as it directly stimulates transcription of the *Mfn1* and *Mfn2* genes [53]. Others have shown that *PGC-1α* stimulates the transcriptional activity of the *Fis1* promoter [26]. We showed that CCG-1423 also significantly reduced the expression level of *PGC-1α,-1* β, *Mfn2,* and *Fis1* (Figure 3C). Presumably, inhibition of the *PGC-1α* gene by CCG-1423 negatively regulated the expression of *Mfn2* and *Fis1*. *PGC-1α* regulates the multiple transcriptional factors involved in mitochondrial biogenesis, such as transcription of the gene coding mitochondrial transcription factor A (TFAM), a gene required for mitochondrial biogenesis [54]. It has been well studied that PGC-1α can also regulate the composition and functions of individual mitochondria [55,56,57]. Thus, CCG-1423 inhibition of PGC-1α could have affected mitochondrial biogenesis and overall function. Mitochondrial oxidative phosphorylation consists of five multienzyme complexes (Complexes I–V) located in the mitochondrial inner membrane [58]. Mitochondrial respiratory complex I is critical to the balance in NAD: NADH levels [59]. CCG-1423 also repressed the gene expression of complex I genes (Figure 3D). Previous studies by our group have also suggested that *NDUFAB1*, a subunit of complex I, interacts with p49/STRAP [24]. Therefore, CCG-1423 inhibition of p49/STRAP may have indirectly altered the gene expression of *NDUFAB1*. The actin cytoskeleton mediated differentiation by regulating SRF transcriptional activity has been well studied [60]. Our data in immunolabeling experiments validate the SRF actin regulation since CCG-1423 affects the actin organization in C2C12 cells (Figure 4A,B). Furthermore, it also affects the mitochondrial membrane potential and mitochondrial structure (Figure 4C,D).

In this study, we investigated the OCR in C2C12 cells after the CCG-1423 treatment, which is associated with reducing mitochondrial respiration (Figure 5A). This inhibitory effect illustrates an impaired mitochondrial function that leads to a reduction of the components of ETC machinery and a decrease in oxidative phosphorylation. Energy consumption from metabolic activities in normal cells relies primarily on mitochondrial oxidative phosphorylation, which is efficient and generates more ATP than glycolysis [61]. The inefficient pathway for energy production by aerobic glycolysis in cancer cells was due to a permanent impairment of mitochondrial OXPHOS [62]. Our data in Figure 5B validates the Warburg concept of this compensatory mechanism. On the treatment of CCG-1423, OXPHOS was inhibited but simultaneously glycolysis was increased as compared to the control at different dosages. Furthermore, total ATP production was greatly reduced with the treatment of CCG-1423 (Figure 5C), which validates the MitoTracker immunolabeling data in Figure 4. We also confirmed the inhibitory effects of CCG-1423 on mitochondrial function with the intact C2C12 cells in high-resolution respirometry, O2k-FluoRespirometer (Figure 6A). The inhibitory effects of CCG-1423 on mitochondrial function are not only limited to C2C12 cells, but we have also tested the inhibitory effects of CCG-1423 on the intact H9C2, a rat cardiomyoblast cell line, using O2k-FluoRespirometer (Supplementary Figure S2). Our data also indicated the importance of CCG-1423 at different complex levels of ETC, as it repressed the activity of ETC in complex I-IV (Figure 6B).

**Figure 7:**
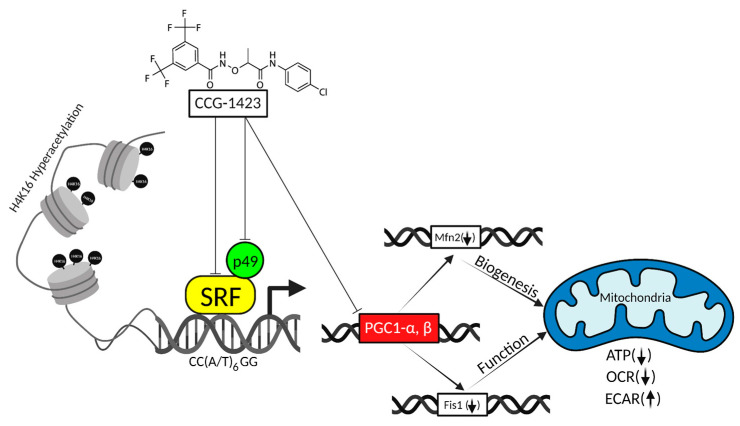
Schematic demonstrating the effects of CCG-1423 on mitochondria function. CCG-1423 inhibits SRF, p49/STRAP and PGC1-α, β, and causes H4K16 hyperacetylation, which modulates the functional activity of oxidative phosphorylation, glycolysis, and total ATP. Arrows indicate: **↑** = increase, ↓ = decrease, and 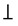 = inhibition.

In conclusion, CCG-1423 inhibits SRF/p49 mediated transcription and contributes to H4K16 hyperacetylation. Most importantly, it blocked the gene expression of *PGC-1α* and *β* and downregulated the mitochondrial genes involved in biogenesis and function. Furthermore, it repressed the mitochondrial oxidative phosphorylation mechanism and reduced total ATP production (Figure 7). Oxidative phosphorylation inhibition has become a potential tool for cancer therapy, and recent therapeutic interventions suggest that some specific tumors might respond to mitochondria inhibitors. Our study supports the effective use of CCG-1423 in repressing mitochondrial genes and function in C2C12 and H9C2 cells representative of vascular and cardiogenic cells. The aging cardiovascular system is vulnerable to damage from oxidative stress and potentially CCG-1423 could be tried to slow down metabolism during periods of high oxidative stress. Although we did not use cancer cell lines in our study, we postulate that the same mechanism of action of CCG-1423 might also prevent cancer cell growth and invasion. However, future studies are required to test the efficacy of this drug as a cardiovascular as well as cancer therapeutic agent.

## 4. Materials and Methods

### 4.1. Cell Culture and Cell Proliferation Assay

The mouse skeletal muscle C2C12 cell line was obtained from the American Type Culture Collection (ATCC, CRL-1772), and all the cell culture reagents were obtained from Thermo Fisher Scientific as previously described [63]. The proliferation ability of C2C12 cells was assessed using (3-(4,5-dimethylthiazol-2-yl)-5-(3-carboxymethoxyphenyl)-2-(4-sulfophenyl)-2H-tetrazolium) (MTS reagent) (Abcam, ab197010). C2C12 cells (5 × 10^3^/well) were seeded in a 96-well microtiter plate with a final volume of 200 μL/well DMEM containing 10% FBS and treated with different concentrations of CCG-1423 (Cayman Chemicals, 10010350) for 24 h. Furthermore, 20 μL of an MTS reagent was added to each well three hours before the end of the incubation period following the manufacturer’s instructions. The absorbance of each well was detected at 490 nm with a microplate reader (BioTek Synergy H1).

### 4.2. Relative Quantification of Gene Expression

The cells were pre-treated for 24 h with a 10 μM dosage of CCG-1423. The isolation of total RNA, cDNA synthesis, and quantification of gene expression were performed as previously described [64]. The sequence of the qPCR primers is described in Supplementary Table S1.

### 4.3. Immunoprecipitation and Western Blot Analysis

Cells were washed with cold phosphate-buffered saline (PBS) and lysed with RIPA lysis buffer system (SantaCruz, sc-24948A) for 10 min at 4 °C after 24 h, 10 µM CCG-1423 treatment. The immunoprecipitation and Western blotting were carried out as previously described [24]. Antibodies that were used included SRF (G-20, SC-335), p49/STRAP antibody [23], PGC-1α (Abcam, 106814), Histone H4 (Cell signaling, L64C1), anti-acetyl-Histone H4 (Lys16) (Cell signaling, E2B8W) and anti-acetyl-Lysine (EMD Millipore, 4G12), anti-goat HRP (Santacruz, sc-20200), anti-mouse HRP (Invitrogen, 62-6520), and anti-rabbit AP (Bio-Rad, 64251130). Immunoreactive bands were visualized with ECL, and images were captured on a ChemiDoc MP Imaging System (Bio-Rad) and analyzed using ImageJ software (National Institutes of Health, Bethesda, MD, USA), and the relative level was obtained by comparison with the GAPDH or total protein level.

### 4.4. MitoTracker and Immunofluorescence Staining

C2C12 cells were seeded with 1.0 × 10^5^ cells per well in a 35 mm MatTek microscopy glass dish (Fisher Scientific, PDCFOS30) and treated with 10 μM of CCG-1423 for 24 h, whereas 0.5% DMSO treatment was used as control. As described previously [24], cells were fixed with 3.7% formaldehyde (Fisher Scientific, SF100-4) for 10 min at room temperature and permeabilized with PBS containing 0.2% Triton X-100 for 5 min. Cells were washed with PBS and blocked with 3% BSA for 1 h at room temperature. Cells were then incubated with ActinGreen (ThermoFisher, Waltham, MA, USA, R37110) using rhodamine-phalloidin (1:200) for 20 min. Samples were then counterstained for 5 min with DAPI (1:1000) to label the nuclei (ThermoFisher, Waltham, MA, USA, D1306). Cells were stained with MitoTracker™ Red CMXRos (Invitrogen, Waltham, MA, USA, M7512) for 30 min following the manufacturer’s instructions. Fluorescent images were captured with a Zeiss LSM 880 confocal microscope with ZEN blue 3.2 software (Carl Zeiss Microscopy, White Plains, NY, USA). The collected images were analyzed, and fluorescence intensity was quantified using ImageJ software.

### 4.5. Measurement of Mitochondrial Oxygen Consumption, Glycolytic and ATP Rate

C2C12 cells were seeded at 12,000 cells/well in XFe96 Well plates (Seahorse Bioscience, Billerica, MA, USA). The cells were treated with different concentrations of CCG-1423 and 0.5% DMSO as control, for 24 h. Then, the cells were subjected to extracellular flux analysis using the XF Cell Mito Stress Test Kit (Agilent, 103015-100), XF Glycolytic Rate Assay Kit (Agilent, 103344-100), and XF Real-Time ATP Rate Assay (Agilent, 103592-100). The measurement was performed as previously described [64,65].

### 4.6. High-Resolution Respirometry

The mitochondrial respiration parameters in the intact C2C12 cells were measured using an Oxygraph 2k (O2k) high-resolution respirometer and processed with DatLab 6.2 software (Oroboros Instruments GmbH, Innsbruck, Austria). Cells were treated with a 10 μM dose of CCG-1423 for 24 h and 0.5% DMSO treatment was used as control. Aliquots of 1 × 10^6^ treated cells were analyzed. Briefly, cells were collected and suspended in 2 mL of Mir05 buffer [66]. Air calibration of a polarographic oxygen sensor was performed routinely before each experiment. According to the O2K manufacturer’s protocol, 5 nM of oligomycin (Sigma, O4876) was added to both chambers. Oligomycin is an inhibitor of ATP synthase and is used to induce a LEAK respiration state of respiration. Then, 0.5–3 µM of CCCP (Sigma, C2759), an uncoupler of mitochondrial oxidative phosphorylation, was added until maximal mitochondrial ET capacity was achieved. Furthermore, 2.5 μM of antimycin A (Sigma, A8674) was added to inhibit the ATP synthase and reduce the OCR. The activity of mitochondrial respiratory complexes was measured according to the substrate-inhibitor-titration protocols adapted as described in [67,68]. First, C2C12 cells (2 × 10^6^) were incubated for 15 min at 4 °C with digitonin (Sigma, D5628) (8 μM/million cells), prepared in MiRO5 buffer to the permeabilize cells. Non-phosphorylating respiration was induced by adding the complex I-linked substrate 2 mM malate (Sigma, M1000) and 10 mM glutamate (Sigma, G1626), followed by 2.5 mM ADP (Sigma, A5285) to achieve state 3 respiration. Subsequently, 10µM of cytochrome C (Sigma, C7752) was added to check the flux control efficiency. Furthermore, 0.5 µM Rotenone (Sigma, R8875) was added to inhibit complex I. Then, 10 mM succinate (Sigma, S2378) was added to evaluate OXPHOS-capacity of complex II and III-linked activity, followed by 1 mM malonic acid (Sigma, M1296) to inhibit complex II respiration and 2.5 µM antimycin A to inhibit complex III. Furthermore, 0.5 mM tetramethyl-p-phenylenediamine, TMPD (Sigma, T3134), and 2 mM ascorbate (Sigma, A7631) to stabilize the TMPD was added to initiate the Complex IV respiration and inhibited using 100 mM Azide (Sigma, 438456). Data analysis was performed with DatLab 6.2 software (Innsbruck, Austria) and cellular respiration of each mitochondrial complex was expressed as oxygen flux (pmol/s*Million Cells).

### 4.7. Statistical Analysis

Statistical analysis was performed with GraphPad Prism 9.1.1 Software Inc (Dotmatics, Boston, MA, USA). Results are represented by mean values ± SD of at least three independent experiments, as indicated by n. For simple comparisons, a two-tailed Student’s *t*-test was used. For multiple group comparison, one way ANOVA was used. *p*-values inferior to 0.05 were considered statistically significant (* *p* < 0.05; ** *p* <0.01; *** *p* < 0.001, **** *p* < 0.0001).

## Data Availability

The raw data are available without reservation upon reasonable request.

## Supplementary Materials

The following supporting information can be downloaded at: https://www.mdpi.com/article/10.3390/ijms231911536/s1.

## References

[1] McDonald O.G., Wamhoff B.R., Hoofnagle M.H., Owens G.K. (2006). Control of SRF binding to CArG box chromatin regulates smooth muscle gene expression in vivo. J. Clin. Investig..

[2] Miano J.M. (2003). Serum response factor: Toggling between disparate programs of gene expression. J. Mol. Cell Cardiol..

[3] Xie L. (2014). MKL1/2 and ELK4 co-regulate distinct serum response factor (SRF) transcription programs in macrophages. BMC Genom..

[4] Esnault C., Stewart A., Gualdrini F., East P., Horswell S., Matthews N., Treisman R. (2014). Rho-actin signaling to the MRTF coactivators dominates the immediate transcriptional response to serum in fibroblasts. Genes Dev..

[5] Miano J.M., Long X., Fujiwara K. (2007). Serum response factor: Master regulator of the actin cytoskeleton and contractile apparatus. Am. J. Physiol. Cell Physiol..

[6] Olson E.N., Nordheim A. (2010). Linking actin dynamics and gene transcription to drive cellular motile functions. Nat. Rev. Mol. Cell Biol..

[7] Miralles F., Posern G., Zaromytidou A.-I., Treisman R. (2003). Actin dynamics control SRF activity by regulation of its coactivator MAL. Cell.

[8] Tsou P.-S., Haak A.J., Khanna D., Neubig R.R. (2014). Cellular mechanisms of tissue fibrosis. 8. Current and future drug targets in fibrosis: Focus on Rho GTPase-regulated gene transcription. Am. J. Physiol. Cell Physiol..

[9] Sakai N., Chun J., Duffield J.S., Wada T., Luster A.D., Tager A.M. (2013). LPA1-induced cytoskeleton reorganization drives fibrosis through CTGF-dependent fibroblast proliferation. FASEB J..

[10] Evelyn C.R., Wade S.M., Wang Q., Wu M., Iñiguez-Lluhí J.A., Merajver S.D., Neubig R.R. (2007). CCG-1423: A small-molecule inhibitor of RhoA transcriptional signaling. Mol. Cancer Ther..

[11] Hayashi K., Watanabe B., Nakagawa Y., Minami S., Morita T. (2014). RPEL proteins are the molecular targets for CCG-1423, an inhibitor of Rho signaling. PLoS ONE.

[12] Jin W., Goldfine A.B., Boes T., Henry R.R., Ciaraldi T.P., Kim E.-Y., Emecan M., Fitzpatrick C., Sen A., Shah A. (2011). Increased SRF transcriptional activity in human and mouse skeletal muscle is a signature of insulin resistance. J. Clin. Investig..

[13] Minami T., Kuwahara K., Nakagawa Y., Takaoka M., Kinoshita H., Nakao K., Kuwabara Y., Yamada Y., Yamada C., Shibata J. (2012). Reciprocal expression of MRTF-A and myocardin is crucial for pathological vascular remodelling in mice. EMBO J..

[14] Johnson L.A., Rodansky E.S., Haak A.J., Larsen S.D., Neubig R.R., Higgins P.D.R. (2014). Novel Rho/MRTF/SRF inhibitors block matrix-stiffness and TGF-β-induced fibrogenesis in human colonic myofibroblasts. Inflamm. Bowel Dis..

[15] Karnkowska A., Vacek V., Zubáčová Z., Treitli S.C., Petrželková R., Eme L., Novák L., Žárský V., Barlow L.D., Herman E.K. (2016). A Eukaryote without a mitochondrial organelle. Curr. Biol..

[16] Guo Y., Jardin B.D., Zhou P., Sethi I., Akerberg B.N., Toepfer C.N., Ai Y., Li Y., Ma Q., Guatimosim S. (2018). Hierarchical and Stage-Specific Regulation of Murine Cardiomyocyte Maturation by Serum Response Factor. Nat. Commun..

[17] Rugowska A., Starosta A., Konieczny P. (2021). Epigenetic modifications in muscle regeneration and progression of duchenne muscular dystrophy. Clin. Epigenet..

[18] Mack C.P. (2011). Signaling mechanisms that regulate smooth muscle cell differentiation. Arterioscler. Thromb. Vasc. Biol..

[19] Arif M., Selvi B.R., Kundu T.K. (2010). Lysine acetylation: The tale of a modification from transcription regulation to metabolism. Chembiochem.

[20] Millar C.B., Kurdistani S.K., Grunstein M. (2004). Acetylation of yeast histone H4 lysine 16: A switch for protein interactions in heterochromatin and euchromatin. Cold Spring Harb. Symp. Quant. Biol..

[21] Schratt G., Philippar U., Berger J., Schwarz H., Heidenreich O., Nordheim A. (2002). Serum response factor is crucial for actin cytoskeletal organization and focal adhesion assembly in embryonic stem cells. J. Cell Biol..

[22] Ellis P.D., Martin K.M., Rickman C., Metcalfe J.C., Kemp P.R. (2002). Increased actin polymerization reduces the inhibition of serum response factor activity by Yin Yang 1. Biochem. J..

[23] Zhang X., Azhar G., Zhong Y., Wei J.Y. (2004). Identification of a novel serum response factor cofactor in cardiac gene regulation. J. Biol. Chem..

[24] Zhang X., Azhar G., Helms S., Zhong Y., Wei J.Y. (2008). Identification of a subunit of NADH-dehydrogenase as a P49/STRAP-binding protein. BMC Cell Biol..

[25] Zhang X., Williams E.D., Azhar G., Rogers S.C., Wei J.Y. (2016). Does P49/STRAP, a SRF-binding protein (SRFBP1), modulate cardiac mitochondrial function in aging?. Exp. Gerontol..

[26] Martin O.J., Lai L., Soundarapandian M.M., Leone T.C., Zorzano A., Keller M.P., Attie A.D., Muoio D.M., Kelly D.P. (2014). A role for peroxisome proliferator-activated receptor γ coactivator-1 in the control of mitochondrial dynamics during postnatal cardiac growth. Circ Res..

[27] Shia W.-J., Pattenden S.G., Workman J.L. (2006). Histone H4 Lysine 16 Acetylation Breaks the Genome’s Silence. Genome Biol..

[28] Esterbauer H., Oberkofler H., Krempler F., Patsch W. (1999). Human peroxisome proliferator activated receptor gamma coactivator 1 (PPARGC1) gene: CDNA sequence, genomic organization, chromosomal localization, and tissue expression. Genomics.

[29] Finck B.N., Kelly D.P. (2006). PGC-1 Coactivators: Inducible regulators of energy metabolism in health and disease. J. Clin. Investig..

[30] Pipes G.C.T., Creemers E.E., Olson E.N. (2006). The myocardin family of transcriptional coactivators: Versatile regulators of cell growth, migration, and myogenesis. Genes Dev..

[31] Gau D., Veon W., Capasso T.L., Bottcher R., Shroff S., Roman B.L., Roy P. (2017). Pharmacological intervention of MKL/SRF signaling by CCG-1423 impedes endothelial cell migration and angiogenesis. Angiogenesis.

[32] Lisabeth E.M., Kahl D., Gopallawa I., Haynes S.E., Misek S.A., Campbell P.L., Dexheimer T.S., Khanna D., Fox D.A., Jin X. (2019). identification of pirin as a molecular target of the CCG-1423/CCG-203971 series of antifibrotic and antimetastatic compounds. ACS Pharmacol. Transl. Sci..

[33] Ma X., Yu H. (2006). Global burden of cancer. Yale J. Biol. Med..

[34] Haga R.B., Ridley A.J. (2016). Rho GTPases: Regulation and roles in cancer cell biology. Small GTPases.

[35] Nakajima M., Hayashi K., Egi Y., Katayama K., Amano Y., Uehata M., Ohtsuki M., Fujii A., Oshita K., Kataoka H. (2003). Effect of Wf-536, a NOVEL ROCK inhibitor, against metastasis of B16 melanoma. Cancer Chemother. Pharmacol..

[36] Wei L., Surma M., Shi S., Lambert-Cheatham N., Shi J. (2016). Novel insights into the roles of Rho kinase in cancer. Arch. Immunol. Ther. Exp..

[37] Zong W.-X., Rabinowitz J.D., White E. (2016). Mitochondria and cancer. Mol. Cell.

[38] Dalton K.M., Lochmann T.L., Floros K.V., Calbert M.L., Kurupi R., Stein G.T., McClanaghan J., Murchie E., Egan R.K., Greninger P. (2021). Catastrophic ATP loss underlies a metabolic combination therapy tailored for *MYCN*-amplified neuroblastoma. Proc. Natl. Acad. Sci. USA.

[39] Wheaton W.W., Weinberg S.E., Hamanaka R.B., Soberanes S., Sullivan L.B., Anso E., Glasauer A., Dufour E., Mutlu G.M., Budigner G.S. (2014). Metformin inhibits mitochondrial complex I of cancer cells to reduce tumorigenesis. eLife.

[40] Roesch A., Vultur A., Bogeski I., Wang H., Zimmermann K.M., Speicher D., Körbel C., Laschke M.W., Gimotty P.A., Philipp S.E. (2013). Overcoming intrinsic multidrug resistance in melanoma by blocking the mitochondrial respiratory chain of slow-cycling JARID1B^high^ cells. Cancer Cell.

[41] Shackelford D.B., Abt E., Gerken L., Vasquez D.S., Seki A., Leblanc M., Wei L., Fishbein M.C., Czernin J., Mischel P.S. (2013). LKB1 inactivation dictates therapeutic response of non-small cell lung cancer to the metabolism drug phenformin. Cancer Cell.

[42] Williams E.D., Rogers S.C., Zhang X., Azhar G., Wei J.Y. (2015). Elevated oxygen consumption rate in response to acute low-glucose stress: Metformin restores rate to normal level. Exp. Gerontol..

[43] Kikuchi K., Shiota J., Yamada T., Ishikawa M., Ihara D., Fukuchi M., Tsuda M., Tabuchi A. (2017). Rho signaling inhibitor, CCG-1423, inhibits axonal elongation and dendritic complexity of rat cortical neurons. Biochem. Biophys. Res. Commun..

[44] Watanabe B., Minami S., Ishida H., Yoshioka R., Nakagawa Y., Morita T., Hayashi K. (2015). Stereospecific inhibitory effects of CCG-1423 on the cellular events mediated by myocardin-related transcription factor A. PLoS ONE.

[45] Gineitis D., Treisman R. (2001). Differential usage of signal transduction pathways defines two types of serum response factor target gene. J. Biol. Chem..

[46] Holst B., Holliday N.D., Bach A., Elling C.E., Cox H.M., Schwartz T.W. (2004). Common structural basis for constitutive activity of the ghrelin receptor family. J. Biol. Chem..

[47] Whitmarsh A.J., Shore P., Sharrocks A.D., Davis R.J. (1995). Integration of MAP kinase signal transduction pathways at the serum response element. Science.

[48] Connelly J.T., Mishra A., Gautrot J.E., Watt F.M. (2011). Shape-induced terminal differentiation of human epidermal stem cells requires P38 and is regulated by histone acetylation. PLoS ONE.

[49] Gräff J., Tsai L.-H. (2013). Histone acetylation: Molecular mnemonics on the chromatin. Nat. Rev. Neurosci..

[50] Alberts A.S., Geneste O., Treisman R. (1998). Activation of SRF-regulated chromosomal templates by Rho-family GTPases requires a signal that also induces H4 hyperacetylation. Cell.

[51] Huang M., Lou D., Charli A., Kong D., Jin H., Zenitsky G., Anantharam V., Kanthasamy A., Wang Z., Kanthasamy A.G. (2021). Mitochondrial dysfunction–induced H3K27 hyperacetylation perturbs enhancers in Parkinson’s disease. JCI Insight.

[52] Kopinski P.K., Janssen K.A., Schaefer P.M., Trefely S., Perry C.E., Potluri P., Tintos-Hernandez J.A., Singh L.N., Karch K.R., Campbell S.L. (2019). Regulation of nuclear epigenome by mitochondrial DNA heteroplasmy. Proc. Natl. Acad. Sci. USA.

[53] Scarpulla R.C. (2011). Metabolic control of mitochondrial biogenesis through the PGC-1 family regulatory network. Biochim. Biophys. Acta (BBA) Mol. Cell Res..

[54] Ekstrand M.I., Falkenberg M., Rantanen A., Park C.B., Gaspari M., Hultenby K., Rustin P., Gustafsson C.M., Larsson N.-G. (2004). Mitochondrial transcription factor A regulates MtDNA copy number in mammals. Hum. Mol. Genet..

[55] Austin S., St-Pierre J. (2012). PGC1α and mitochondrial metabolism—Emerging concepts and relevance in ageing and neurodegenerative disorders. J. Cell Sci..

[56] Wu Z., Puigserver P., Andersson U., Zhang C., Adelmant G., Mootha V., Troy A., Cinti S., Lowell B., Scarpulla R.C. (1999). Mechanisms controlling mitochondrial biogenesis and respiration through the thermogenic coactivator PGC-1. Cell.

[57] Chen L., Qin Y., Liu B., Gao M., Li A., Li X., Gong G. (2022). PGC-1α-Mediated Mitochondrial Quality Control: Molecular Mechanisms and Implications for Heart Failure. Front. Cell Dev. Biol..

[58] Hatefi Y. (1985). The mitochondrial electron transport and oxidative phosphorylation system. Annu. Rev. Biochem..

[59] Santidrian A.F., Matsuno-Yagi A., Ritland M., Seo B.B., LeBoeuf S.E., Gay L.J., Yagi T., Felding-Habermann B. (2013). Mitochondrial complex I activity and NAD+/NADH balance regulate breast cancer progression. J. Clin. Investing..

[60] Connelly J.T., Gautrot J.E., Trappmann B., Tan D.W.-M., Donati G., Huck W.T.S., Watt F.M. (2010). Actin and serum response factor transduce physical cues from the microenvironment to regulate epidermal stem cell fate decisions. Nat. Cell Biol..

[61] Koppenol W.H., Bounds P.L., Dang C.V. (2011). Otto Warburg’s Contributions to Current Concepts of Cancer Metabolism. Nat. Rev. Cancer.

[62] Zheng J. (2012). Energy metabolism of cancer: Glycolysis versus oxidative phosphorylation (review). Oncol. Lett..

[63] Rogers S.C., Zhang X., Azhar G., Luo S., Wei J.Y. (2013). Exposure to high or low glucose levels accelerates the appearance of markers of endothelial cell senescence and induces dysregulation of nitric oxide synthase. J. Gerontol. Ser. A.

[64] Zhang X., Ameer F.S., Azhar G., Wei J.Y. (2021). Alternative splicing increases sirtuin gene family diversity and modulates their subcellular localization and function. IJMS.

[65] Zhang X., Azhar G., Wei J.Y. (2012). The expression of MicroRNA and MicroRNA clusters in the aging heart. PLoS ONE.

[66] Gnaiger E. (2020). Mitochondrial pathways and respiratory control. An Introduction to OXPHOS Analysis.

[67] Tobacyk J., Kc G., MacMillan-Crow L.A. (2021). Overexpression of MnSOD protects against cold storage-induced mitochondrial injury but not against OMA1-Dependent OPA1 proteolytic processing in rat renal proximal tubular cells. Antioxidants.

[68] Pesta D., Gnaiger E., Palmeira C.M., Moreno A.J. (2012). High-resolution respirometry: OXPHOS protocols for human cells and permeabilized fibers from small biopsies of human muscle. Mitochondrial Bioenergetics.

