# Supplementary material for: Rho/SRF Inhibitor Modulates Mitochondrial Functions
Source: Int J Mol Sci. 2022 Sep 29;23(19):11536. doi: 10.3390/ijms231911536 (PMC9570101; doi:10.3390/ijms231911536)
Supplement: Supplementary file 1 [file ijms-23-11536-s001.zip › ijms-1917366-supplementary.pdf]

Supplementary Data

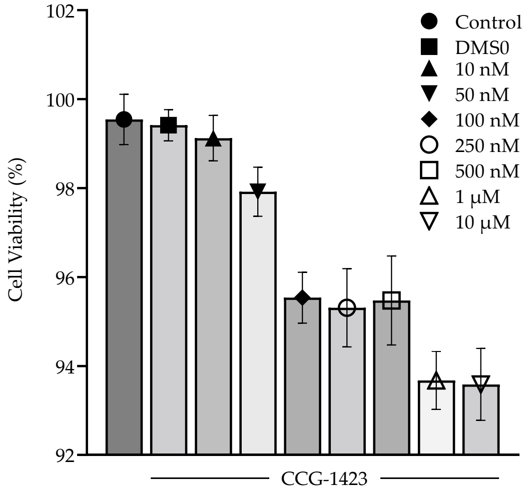

Supplementary Figure S1. MTS assay was performed on C2C12 cells treated with vehicle or different concentrations of CCG-1423 (10 nM - 10 μM) for 24 h, which showed, that CCG-1423 does not affect on the cell viability.

A

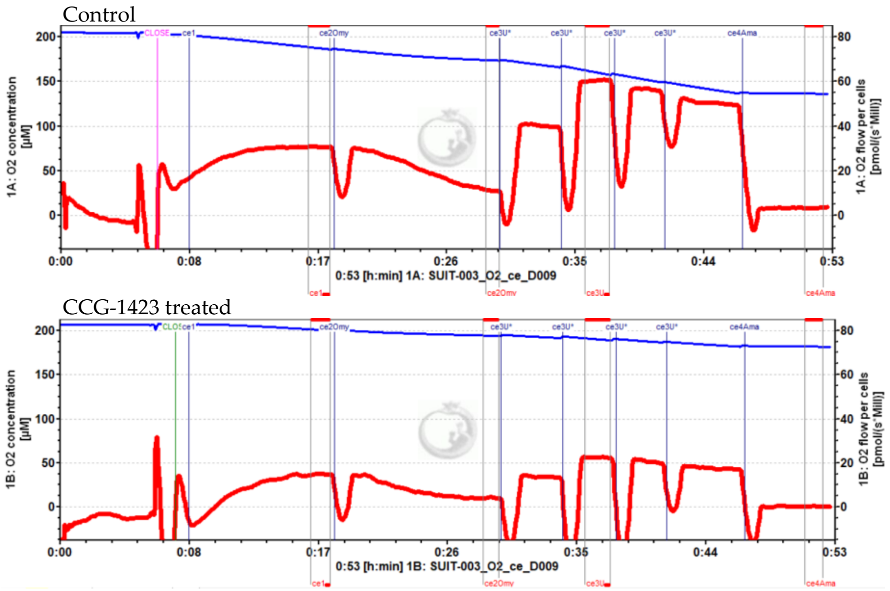

B

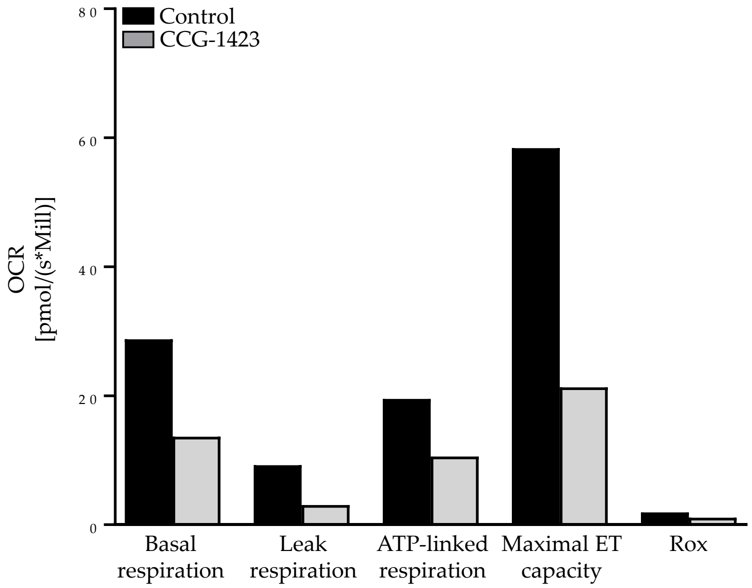

Supplementary Figure S2. High-resolution respiratory analysis of oxidative phosphorylation A) Representative images of Oxygraph at O2K of intact H9C2 cells treated with CCG-1423 with 10 μM for 24h. B) Oxygen consumption rate (OCR) and basal respiration, leak respiration, ATP-linked respiration, Maximum ET capacity was analyzed after the treatment of CCG-1423 and compared with non-treated H9C2 cells in Oroboros O2K instrument.

Supplement Table S1. List of primer sequences used for qPCR analysis.

Name	Primer Sequence (5' - 3')
SRF-F	CTACACGACCTTCAGCAAGAG
SRF-R	GTATACACATGGCCTGTCTCAC
p49/STRAP-F	GGGACTCTGAACCTCAATAACG
p49/STRAP-R	ACACTCCTGACAAGTTTTCGG
PGC-1α-F	ATTCGGGAGCTGGATGGCTT
PGC-1α-R	AGCAGCACACTCTATGTCACTC
PGC-1β –F	CAGGGTGGGGACTCTGGA
PGC-1β –R	AGTCAAAGTCACTGGCGTCC
MFN1-F	ACCCCATGAAGACTAGGCGA
MFN1-R	ACCCCATGAAGACTAGGCGA
MFN2-F	GGACAGCCACTTAGGGAACC
MFN2-R	GTCCTGGTAGGCAAAGACCC
Fis1-F	CGCTACGGAGAAAAACCAAAGA
Fis1-R	TTCAAAATTCCTTGCAGCTTCG
Opa1-F	TACTGTTAGCCCCGAGACCA

Opa1-R	GATGACACCAGGCAAGTCCA
NDUFV1-F	AAGCCATCGCTCGTCTCAT
NDUFV1-R	TTCATCCAGTCAACGCCCTC
NDUFV2-F	TCFFTTTGCTTATTCCCACCT
NDUFV2-R	GACCGAGATAGTGGTCCTGT
NDUFAB1-F	ACATTGCAGATAAGAAGGATGTGT
NDUFAB1-R	TCACTCTTGGTTGTCAGTGTGT
NDUFS1-F	CAGGGGCTGCTTACACAGAA
NDUFS1-R	AGGCGTCACTGCTACTTTGG

F, forward primer; R, reverse primer.
